# GPCR oligomers in pharmacology and signaling

**DOI:** 10.1186/1756-6606-4-20

**Published:** 2011-05-27

**Authors:** Javier González-Maeso

**Affiliations:** 1 Departments of Psychiatry and Neurology, Friedman Brain Institute, Mount Sinai School of Medicine, New York, New York, USA

## Abstract

G protein-coupled receptors (GPCRs) represent one of the largest families of cell surface receptors, and are the target of more than half of the current therapeutic drugs on the market. When activated by an agonist, the GPCR undergoes conformational changes that facilitate its interaction with heterotrimeric G proteins, which then relay signals to downstream intracellular effectors. Although GPCRs were thought to function as monomers, many studies support the hypothesis that G protein coupling involves the formation of GPCR homo- and/or hetero-complexes. These complex systems have been suggested to exhibit specific signaling cascades, pharmacological, internalization, and recycling properties. In this review, we summarize recent advances in our understanding of the structure, function and dynamics of GPCR complexes, as well as the findings obtained in animal models.

## Introduction

G protein-coupled receptors (GPCRs) [1], also known as seven transmembrane receptors, were initially considered to function as monomeric structural units. In the last decade, however, there has been an increasing number of publications describing GPCR homodimers or even higher order oligomers [2-5]. There is also evidence that GPCR heterocomplexes may exist [6-8]. Two of the main lines of research in structure and function of GPCRs are focused on the residues and domains responsible for GPCR complex formation, as well as the stability and mechanisms of signaling crosstalk between the components of these homo- and heteromers. Another point of interest is the identification of GPCR complexes in native tissues and their functional significance in whole animal models. In this review, we will summarize recent advances in our understanding of GPCR homo- and heteromerization, as well as their potential implication in physiological responses.

## History of GPCR complexes

In the 1980s, two laboratories at the Karolinska Institute in Stockholm and the National Institute for Medical Research in London proposed the existence of receptor mosaics [9,10] and a direct interaction of two receptors with each other [11]. This hypothesis was also supported by previous publications demonstrating negatively cooperative site-site interactions among the β-adrenergic receptors [12]. Similarly, using a photoaffinity labeling approach, Sokolovsky and collaborators proposed that muscarinic receptors exist in interconvertible dimer and tetramer forms [13]. They also suggested a model in which muscarinic receptor monomers formed dimers and tetramers joined by covalent bonds; although this model of covalent bonding has not been confirmed. Immunoaffinity chromatography approaches with the β_2_-adrenergic receptor in mammalian lung [14], and protocols of radiation inactivation with the α_2_-adrenergic receptor in human platelets [15] also pointed toward the phenomenon of GPCR oligomerization. Years earlier, it had been introduced the concept that antagonists bind to the receptor with one uniform affinity whereas, for agonists, two distinct binding sites of high and low affinity are detected [16]. They demonstrated that the proportion of agonist high affinity binding sites was dramatically decreased in the presence of guanine nucleotides, and proposed the ternary complex model to explain these findings [17]. In the 1990s, additional studies based on radioligand binding displacement curves obtained with muscarinic [18] and dopamine D2 [19] receptors were consistent with the idea that the tested ligands differentially bind monomeric and dimeric GPCRs. Potter and colleagues found that competition curves between different agonists and antagonists with the muscarinic receptor antagonist N-[^3^H]methylscopolamine ([^3^H]NMS) showed the presence of equal populations of guanine nucleotide-sensitive high affinity sites (H) and guanine nucleotide-insensitive low affinity sites (L) in membrane preparations from rat brainstem [18]. These two binding sites could be blocked independently using irreversible ligands, and the specific blockage of either H or L sites did not lead to changes to the remaining binding sites. This phenomenon, which the authors termed 50% H, was interpreted as evidence of M2 muscarinic receptor dimerization. Seeman and collaborators compared the binding properties of the dopamine D2 antagonists [^3^H]emonapride and [^3^H]spiperone in tissue cultures and in postmortem human caudate nucleus [19]. They found that the density of D2 receptors labeled with [^3^H]emonapride was twice higher than that labeled with [^3^H]spiperone. Similar findings were obtained by positron emission tomography (PET) in human brain when using [^11^C]raclopride and [^11^C]spiperone [20]. They proposed that [^3^H]spiperone preferentially binds dopamine D2 receptor dimers, whereas [^3^H]emonapride only binds to receptor monomers. These and other observations led the authors to speculate that GPCRs might be expressed and function as dimers or oligomers. However, the existence of GPCR oligomeric structures did not attract much attention, and the most accepted model was the one proposing that a single GPCR is sufficient to interact with a single G protein.

It was not until the findings by Wess that the concept of GPCR complex formation gained interest among the research field [21]. They tested the functional response induced by chimeras of the muscarinic M3 and α_2C_-adrenergic receptors. Chimeric M3/α2C receptor was composed of the first five transmembrane domains (including intracellular loop 3) of the muscarinic M3 receptor and the last two transmembrane domains of the α_2C_-adrenergic receptor. Chimeric α2C/M3 receptor was composed of the first five transmembrane domains α_2C_-adrenergic receptor and the last two transmembrane domains (including intracellular loop 3) of the muscarinic M3 receptor.

Intracellular loop 3 of the muscarinic M3 receptor was maintained unchanged in both chimeras because this region is responsible for the specificity of G protein coupling. The signaling induced by the muscarinic receptor agonist carbachol was abolished when either of these two chimeras was expressed alone. However, coexpression of M3/α2C and α2C/M3 receptor chimeras rescued the carbachol-induced signaling, which provided the first indirect evidence for GPCR dimerization.

Two of the key findings to indicate that GPCR complexes might be important in receptor signaling were also the assembly of GABA_B _receptor subtypes (GABA_B_-R1 and GABA_B_-R2) into heteromeric complexes (see [22] for review), and the heteromerization of δ-and κ-opioid receptors affecting ligand binding and receptor signaling [23]. Since then, several laboratories have reported the presence of GPCRs as homo- and heteromers, a concept that it is now widely accepted.

## Protein domains responsible for GPCR complex formation

Much evidence indicates that class C GPCRs, including GABA_B _and metabotropic glutamate (mGlu) receptors, exist and function as constitutive dimers. Heterodimerization of GABA_B _receptor is mediated by parallel coiled-coil interactions at C-termini [24-26]. Co-expression of GABA_B_-R1 and GABA_B_-R2 results in the functional expression of GABA_B _receptor in the plasma membrane [22]. Recently, it has also been shown that the mGlu receptors are assembled into strict dimers covalently bound via a disulfide bridge [27]. Closure of both binding domains is necessary to obtain full activity of the mGlu receptors [28]. In contrast, most of the experiments conducted on class A GPCRs suggest that receptor oligomers are formed through non-covalent interactions, with the transmembrane (TM) domains as the most likely elements involved in receptor oligomerization. We will summarize our current knowledge regarding the TM domains that form the interface of GPCR homo- and hetero-complexes.

Milligan and colleagues demonstrated using co-immunoprecipitation and fluorescence resonance energy transfer (FRET) assays that the α_1b_-adrenergic receptor forms oligomeric complexes in which symmetrical contact points involving both TM1 and TM4 were detected [29]. These experiments were performed using epitope-tagged fragments comprising distinct TM domains. They also found with the use of three-color FRET (3-FRET) that the α_1b_-adrenergic receptor is able to form oligomeric complexes comprising at least three monomers [30]. The 3-FRET signal was significantly reduced by a combination of single point mutations of hydrophobic residues in TM4, findings that were also associated with protein maturation and signaling function.

A similar conclusion has been reached with the dopamine D2 receptor. Thus, cysteine cross-linking experiments provided evidence for the involvement of TM1 and TM4 at the symmetrical interface of the dopamine D2 receptor oligomer, and the cross-linking was differentially affected by the presence of dopamine D2 agonists or inverse agonists [31,32]. The combination of bioluminescence/fluorescence complementation and energy transfer approaches also suggested that the dopamine D2 receptor form higher order oligomers in the plasma membrane [33]. They propose a dopamine D2 receptor oligomer with a symmetric interface formed by TM1 and the cytoplasmic helical domain (H8) in addition to the TM4 interface.

Serotonin 5-HT_2A _receptor and mGlu2 receptor form a functional heterocomplex that has been implicated in schizophrenia and psychosis [34,35]. The differences in the capacity of the mGlu2 and mGlu3 receptors to exist as a receptor complex with the 5-HT_2A _receptor provided the basis to identify the specific mGlu2 receptor TM domains responsible for GPCR heterocomplex formation [34]. Study of a series of molecular chimeras suggested that the segment containing TM4 and TM5 of the mGlu2 receptor was both necessary and sufficient for complex formation with the 5-HT_2A _receptor. The mGlu3 receptor chimera containing only this segment from the mGlu2 receptor (mGlu3ΔTM4-5) was capable of co-immunoprecipitating and maintaining close proximity with the 5-HT_2A _receptor as indicated by FRET. In contrast, the chimera mGlu2 ΔTM4-5 did not show evidence of complex formation with the 5-HT_2A _receptor.

Although all these findings support a contribution of TM4 and TM1 to the formation of GPCR complexes, the discrete structural components that affect this phenomenon have not been unraveled.

## Stability of GPCR oligomers

One question to be solved is the stability and lifetime of the GPCR complexes in the plasma membrane. Although GPCR oligomerization has been proposed to be necessary for effective receptor maturation and surface delivery [30,36], recent findings also suggest that GPCRs exist in a dynamic equilibrium between monomeric and dimeric/oligomeric states. Thus, using a fluorescence recovery after photobleaching (FRAP) approach, Bunemann and collaborators demonstrated that the β_1_-adrenergic receptor shows transients interactions on a timescale of seconds [37]. However, for the β_2_-adrenergic receptor, they found stable oligomers in HEK293 cells and cardiac myocytes. Similar findings using FRAP were obtained in studies examining the stability of dopamine D2 receptors, with which they demonstrated a dynamic equilibrium between monomer and homodimer/oligomer states of the dopamine D2 receptor [38].

The dynamic nature of dimer formation of muscarinic M1 receptor has been recently detailed by using total internal reflection fluorescence microscopy (TIRFM) in living cells [39]. They visualized single muscarinic M1 receptors and found that they exist as monomers and dimers, but not oligomers. They also found that ~30% of the receptor molecules exist as a dimer at any given time, and suggested that muscarinic M1 receptors do not form constitutive dimers.

## Signaling crosstalk

Several studies have demonstrated that GPCR heteromerization affects the pharmacological and cellular signaling responses as compared to the different receptor subtypes expressed alone. One well-studied example is the receptor heterocomplex between the α_2a_-adrenergic receptor and the μ-opioid receptor. α_2a_-Adrenergic receptor and μ-opioid receptor have been repeatedly involved in depression and opioid addiction [40-44]. Direct interaction between α_2a_-adrenergic and μ-opioid receptors was demonstrated in tissue cultures and primary neurons [45]. Using this GPCR heterocomplex as a model, Vilardaga and Lohse reported that α_2a_-adrenergic and μ-opioid receptors communicate one with each other through the receptor complex [46]. They found that morphine binding to the μ-opioid receptor induces structural changes in the α_2a_-adrenergic receptor when this is bound to norepinephrine. Previous findings showed that whereas activation of either α_2a_-adrenergic receptor or μ-opioid receptor increases phosphorilation of ERK 1/2 in HEK293 cells and in primary spinal cord neurons, activation of both receptors leads to a decrease signal activation [45]. Using FRET microscopy, it was found that the conformational changes induced in the α_2a_-adrenergic receptor by morphine through the receptor heterocomplex inhibit G protein coupling and the ERK signaling pathway [46].

**Figure 1:**
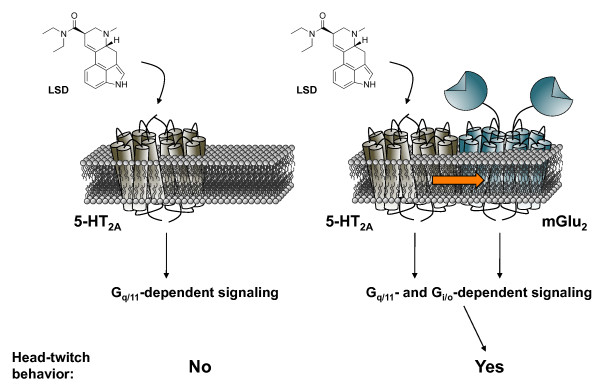
**G protein-dependent signaling and behavioral responses that require a GPCR heterocomplex**. Hallucinogenic drugs, such as lysergic acid diethylamide (LSD), mescaline and psilocybin, all have in common a high affinity for the serotonin 5-HT_2A _receptor. Metabotropic glutamate 2 receptor (mGlu_2_) and serotonin 5-HT_2A _receptor form a specific functional GPCR heterocomplex in mammalian brain and in tissue culture preparations. mGlu_2 _transmembrane domains 4/5 mediate association with the 5-HT_2A _receptor. When 5-HT_2A _and mGlu_2 _are prevented from forming a receptor heterocomplex, activation of 5-HT_2A _by LSD elicits signaling characteristic of G_q/11_-protein subtypes. In contrast, LSD acting at the 5-HT_2A_-mGlu_2 _receptor heterocomplex activates both G_q/11_- and G_i/o_-dependent signaling. Head-twitch behavior is reliably and robustly elicited by hallucinogenic 5-HT_2A _agonists, and is absent in mGlu_2 _knockout mice.

Another indication of GPCR heterocomplexes that affect G protein coupling comes from studies on interactions between 5-HT_2A _and mGlu2 receptors. The 5-HT_2A _receptor and the mGlu2 receptor are usually coupled to G_q/11 _and G_i/o _proteins, respectively. We recently reported that hallucinogenic 5-HT_2A _receptor agonists activate both G_q/11 _and G_i/o _proteins only when the 5-HT_2A _receptor forms a receptor heterocomplex with the mGlu2 receptor [34,47]. These findings have been further supported *in vivo *in murine models. The head-twitch is a behavioral response that is reliably elicited by hallucinogens, and is absent in 5-HT_2A_-KO mice [47]. We found that head-twitch response is abolished in mGlu2-KO mice [48], which suggests that the 5-HT_2A_-mGlu2 receptor complex is necessary for the behavioral responses induced by hallucinogenic 5-HT_2A _receptor agonists (Figure 1).

Although these and other findings support the functional and behavioral significance of GPCR heterocomplexes, many others demonstrate functional interactions between GPCR subtypes that do not require formation of GPCR complexes. It was reported that the GABA_B _receptor potentiates the mGlu1 receptor-dependent signaling, yet these two receptors do not associate with each other [49]. Similarly, it has been shown that the corticotropin-releasing factor 1 (CRF1) receptor modulates G protein coupling and anxiety-like behaviors by sensitizing 5-HT_2A _receptor-dependent signaling [50]. The mechanism of interaction between CRF1 and 5-HT_2A _receptors is not direct, and requires scaffolding proteins.

## GPCR oligomers in native tissues

All the findings described above suggest the existence of GPCR homo- and hetero-complexes in tissue cultures. Similar findings have been obtained with GPCRs reconstituted in lipid vesicles [51]. A fundamental question to be addressed is the presence of GPCR complexes in native tissue. Three recent publications using different approaches support this hypothesis.

Reproduction in animals is dependent on the pulsatile release of pituitary gonadotropic hormones: luitenizing hormone (LH) and follicle-stimulating hormone (FSH). In the testis, LH acts through LH receptors (LHR) on the Leydig cells to regulate the production and secretion of androgens. Biological actions of LH and FSH also include maturation of testis and ovary [52]. Huhtaniemi and colleagues developed transgenic mice expressing mutant LH receptors (LHR) in the absence of functional endogenous receptor (*i.e*., in LHR-KO mice) [53]. They selected two mutant LHRs that were not able to bind the ligand or to modulate G protein coupling by agonist activation. The first mutant LHR (Cys22Ala) is expressed on the cell surface but is incapable of binding the LHR agonist hCG [54]. The second mutant harbors a deletion (Val553 to Ala689) that abolishes G protein coupling and cellular signaling [55]. They confirmed in tissue cultures that the first mutant (LHR^LH-^) did not bind hCG and that the second mutant (LHR^cAMP-^) did not activate cAMP production. Co-expression of LHR^LH- ^and LHR^cAMP- ^partially rescued the cAMP response to hCG stimulation in HEK293 cells [53]. Similar findings for agonist binding and G protein coupling were also observed in the testes of LHR^LH- ^and LHR^cAMP- ^mutant mice. They also found that both transgenic mice showed a phenotype similar to that of LHR-KO mice. This phenotype was rescued in mice expressing both LHR^LH- ^and LHR^cAMP- ^receptors. The testes were normally developed, and transgenic mice expressing both LHR^LH- ^and LHR^cAMP- ^showed serum hormone levels comparable to those seen in wild type mice. The authors proposed intermolecular cooperation *in vivo *as a potential mechanism to understand the physiological relevance of these findings, and suggested GPCR oligomerization as the biophysical basis of these specific interactions.

Co-immunoprecipitation assays represent one of the most commonly used approaches to validate *in vivo *the significance of findings with GPCR heterocomplexes obtained in tissue cultures. This assay suggests that GPCRs might form heterocomplexes in native tissue. Another possibility is that GPCRs form part of the same protein complex, yet they interact indirectly through scaffolding proteins, and are not in close proximity at the plasma membrane. An essential tool to better define the existence of close proximity is the development of antibodies that only react when the two components of the GPCR heterocomplex are co-expressed in the same cell, and not when they are expressed alone. Using a strategy of subtractive immunization, Devi and collaborators developed monoclonal antibodies to selectively recognize the μ-δ opioid receptor heteromer, but not either μ-opioid receptor or δ-opioid receptor, in the central nervous system [56]. They detected μ-δ opioid receptor heteromers in cortical membranes of wild type mice, using μ-KO, δ-KO and μ-δ-KO mice as negative controls. Chronic, but not acute, treatment with morphine induced an increase in the expression of μ- and δ-opioid receptors as a GPCR heterocomplex in brain regions involved in drug addiction such as frontal cortex, striatum and nucleus accumbens.

An interesting approach is the use of FRET with fluorescent ligands in native tissue. Albizu and Durroux optimized the experimental conditions to perform homogenous time-resolved FRET (HTRF) experiments with the use of fluorescent ligands [57]. HTRF is based on the derivation of GPCR ligands with a lanthanide and a compatible fluorophore. The authors previously generated fluorescent ligands for vasopressin and oxytocin receptors [58], which are highly expressed in mammary glands. Using fluorescent ligands, they obtained FRET signal with native oxytocin receptors in rat mammary glands [57]. Experiments revealing GPCR complexes were performed both in membrane preparations and in patches of native tissues. Taken together, these findings suggest that GPCR are placed in close proximity *in vivo *in murine models.

## Conclusions and Future Directions

The functional crosstalk observed in between the components of GPCR heterocomplexes provides a unique scenario for the rational design of new therapeutic drugs. Examples of this include the 5-HT_2A_-mGlu2 receptor complex in schizophrenia [34], the μ-opioid-α_2a_-adrenergic receptor complex in depression and opioid addiction [45], the adenosine A_2A_-dopamine D2 receptor complex in Parkinson's disease [59], and the κ-δ-opioid receptor complex in analgesia and drug abuse [23]. However, the majority of findings suggesting the expression of these GPCRs as homo- and hetero-oligomers have been reported using biochemical, biophysical and pharmacological approaches in tissue cultures, and not in rodents. Only recently we have started to make progress toward the ultimate goal of demonstrating the physiological and behavioral relevance of GPCR heterocomplexes in whole animal models. A tempting approach to validate their significance is the use of knock-in mice that express two GPCRs as functional homomers, but not as heteromers.

Unraveling the residues and domains that form part of the GPCR complex interface might provide the basis for the design of drugs that specifically affect the signaling crosstalk between the components of the receptor complex. Most of the findings obtained in tissue cultures support the hypothesis that TM1 or TM4 and 5 form the interfaces between GPCR monomers to form higher order oligomers. However, crystal structures of the CXCR4 chemokine receptor provide evidence for a symmetric dimer in which monomers interact only at the extracellular side of TM5 and TM6 [60]. These findings are different from previous observations, and make clear the need for further research in the field. Interestingly, the crystallization of rhodopsin revealed an antiparallel dimer with respect to the plane of the membrane [61], most probably due to the effects of solubilization protocols on the physiologically relevant dimers.

Recent breakthroughs in the determination of the crystal structures of GPCRs have provided indirect information regarding G protein coupling. Comparison of the inactive and active [63] structures of the β_2_-adrenergic receptor suggests that the differences are mainly induced at the cytoplasmic region of the receptor, with outward displacement of TM5 and TM6 and inward movement of TM7 and TM3 in the active relative to the inactive structure. In contrast, only small differences were observed between the two protein structures in the agonist-binding pocket. Heterotrimeric G proteins are composed of three subunits: Gα, Gβ and γ [64]. Recent findings based on systematic cross-linking experiments have identified many of the contact sites between muscarinic M3 receptor and Gαq subunit [65]. In addition, Gβ and γ also play a key role in determining receptor-G protein specificity [66]. Since simultaneous interaction of the receptor with both Gα and Gβγ subunits is required for G protein activation, and based on the size of the intracellular region of the receptor exposed to the cytoplasm, it is tempting to speculate that a single receptor might show limited coupling to the heterotrimeric G protein. Although this hypothesis is supported by the development of a functional complementation assay that points to two receptors and a single G protein as the minimal signaling unit of the dopamine D2 receptor [67], it has also been shown that rhodopsin [68] and the β_2_-adrenergic receptor [69] signal efficiently to G proteins when reconstituted into lipid nanodiscs containing a single receptor. Further studies will be needed to better understand the complexity of receptor-G protein coupling [70,71], and the role of GPCR homo-and hetero-complexes in receptor signaling. Despite the advances in our knowledge of structure and function of GPCRs, much more needs to be learned about the pharmacology, neuroanatomy and signaling of GPCR oligomers both *in vitro *and in mouse models before developing new therapeutic drugs that affect their function.

## Competing interests

The authors declare that they have no competing interests.

## Authors' contributions

JGM searched the literature, conceived the topic of the review and wrote the manuscript
